# Notes and Comments

**Published:** 1893-10

**Authors:** 


### Notes and Comments.[1]

An Interesting Case.—A young lady, about seventeen years of age, called for treatment. She was suffering from severe pain in the region of the right superior lateral incisor. In her own words, "suffering from neuralgia in the front teeth on the right side," and wished the lateral incisor extracted, as it was loose and had caused her trouble enough, etc. Upon examination we found the teeth in remarkably good condition for a girl of this age, excepting the tooth mentioned, it being slightly loosened; we also found a slight bluish line running through the gum over the root of this tooth and part way across the adjoining central incisor. Upon inquiry as to her ever having received a blow or a fall injuring these parts, we learned from her mother that about five years previous to her

[1] The assistant editor solicits contributions for this department,—new methods, new remedies and formulas, or any short practical note which may prove of value to the practitioner or student. Address 1506 Arch Street, Philadelphia.

visit she fell, striking the parts upon the point of a lead-pencil; but both the girl and her mother, in response to our questions, felt sure that the pencil was all drawn out at the time. However, after some persuasion, the patient allowed us to make an incision and explore the parts.

We found the lower border of the process absorbed over the root of the lateral incisor, and a small piece of the pencil lying directly over the root; also another piece, possibly a quarter of an inch in length, that had been forced endwise between the central and lateral incisors. It was so tightly embedded that we did not remove it in one piece, but found it necessary to dissect it out, a small piece at a time. The wound was then dressed and the patient dismissed. She was not heard from for several weeks, when the report was made that "the loose tooth had grown tight and the neuralgia had disappeared."

---

Business and Professional Methods.—In a very practical paper, published in the *Dental Register*, Dr. C. B. Blackmarr urges the importance of conducting one's practice in a business-like way. He says, in speaking of a fellow-practitioner, a most excellent and conscientious operator, that it was pitiful to see him try to make his income pay expenses, and as he is growing older the struggle is growing harder. He goes about his work in about this manner: he will examine the tooth several times with mirror and explorer, and talks to the patient long enough about what would have to be done under certain circumstances, etc., for many operators to perform the operation. Then in making an appointment, he says, "Come at half-past one or two," and as he was not exact in regard to his time, the patient was not in coming, and came at nearly half-past two. The tooth was filled and finished about half-past four,—too late to wait upon another patient. Then, upon being asked the amount of his fee, he reasoned this way: "One hour's consultation and two hours' operating." And yet he cannot imagine why, in a business sense, his professional career is a failure.

We are familiar with numerous cases somewhat similar, where the man or woman fails to combine business methods with professional principles. This case is cited here, however, as a hint, not only to students and the younger members of the profession, but to some who have been plodding for years and are still wondering why they fail to succeed.

